# Ofatumumab Monoclonal Antibody Affinity Maturation Through *in silico* Modeling

**DOI:** 10.22034/ibj.22.3.180

**Published:** 2018-05

**Authors:** Zahra Payandeh, Masoumeh Rajabibazl, Yousef Mortazavi, Azam Rahimpour, Amir Hossein Taromchi

**Affiliations:** 1 Department of Medical Biotechnology and Nanotechnology, Faculty of Medicine, Zanjan University of Medical Sciences, Zanjan, Iran; 2 Department of Clinical Biochemistry, Faculty of Medicine, Shahid Beheshti University of Medical Sciences, Tehran, Iran; 3 School of Advanced Technologies in Medicine, Shahid Beheshti University of Medical Sciences, Tehran, Iran; 4 Cancer Gene Therapy Research Center, Faculty of Medicine, Zanjan University of Medical Sciences, Zanjan, Iran

**Keywords:** Monoclonal antibody, Ofatumumab, Protein engineering

## Abstract

**Background:**

Ofatumumab, an anti-CD20 mAb, was approved in 2009 for the treatment of chronic lymphocytic leukemia. This mAb acts through immune-mediated mechanisms, in particular complement-dependent cytotoxicity and antibody-dependent cellular cytotoxicity by natural killer cells as well as antibody-dependent phagocytosis by macrophages. Apoptosis induction is another mechanism of this antibody. Computational docking is the method of predicting the conformation of an antibody-antigen from its separated elements. Validation of the designed antibodies is carried out by docking tools. Increased affinity enhances the biological action of the antibody, which in turn improves the therapeutic effects. Furthermore, the increased antibody affinity can reduce the therapeutic dose of the antibody, resulting in lower toxicity and handling cost.

**Methods:**

Considering the importance of this issue, using *in silico* analysis such as docking and molecular dynamics, we aimed to find the important amino acids of the Ofatumumab antibody and then replaced these amino acids with others to improve antibody-binding affinity. Finally, we examined the binding affinity of antibody variants to antigen.

**Results:**

Our findings showed that variant 3 mutations have improved the characteristics of antibody binding compared to normal Ofatumumab antibodies.

**Conclusion:**

The designed anti-CD20 antibodies showed potentiality for improved affinity in comparison to commercial Ofatumumab.

## INTRODUCTION

Monoclonal antibodies (mAbs) are the most widely used and effective biological drugs for “targeted therapy” of cancer[1], which can also serve as extremely relevant diagnostic and biotechnological tools[2,3]. Based on Food and Drug Administration (FDA) latest reports, there are currently 46 approved therapeutic mAbs in the market in the United States or Europe as well as over 100 antibody candidates in clinical development[4]. Antibodies can function as ‘magic bullets’ in cancer treatment because they have special ability in identifying a large number of specific epitopes and high-affinity binding to different types of antigen[5]. The high specificity and binding affinity of antibodies to antigens enable them to be used as therapeutic agents for treating different diseases[6]. Therapeutic antibodies have certain advantages over small molecules or other protein therapeutics such as longer serum half-lives, higher avidity and selectivity, and the ability to invoke desired immune responses[7]. Antibodies are also organized into distinct structural and functional domains, which have facilitated their engineering[8].

Since the first introduction of humanized IgG1 antibodies to the market in the late 1990s, different kinds of engineering have been performed on IgG antibody molecules. When designing the therapeutic antibodies, different features of these molecules, including binding affinity, tissue penetration, immunogenicity, stability, effector functions, and antibody half-life should be considered.

One of the most extensively studied areas of antibody engineering is affinity maturation or improvement of the antigen-binding affinity. Increased affinity enhances the biological activity of the antibody, which in turn improves the therapeutic outcomes. Furthermore, the increased antibody affinity can reduce the therapeutic dose of antibody, resulting in lower toxicity and treatment cost[9-11].

Different technologies are available for enhancing antibody affinity, including *in vivo* methods in mammalian immune system and several *in vitro* approaches. Although effective, these methods are time-consuming, cannot target a specific epitope and are unable to tolerate rapid changes of antigens[12]. Hence, advances in antibody design technology and a deeper understanding of the interaction of therapeutic antibodies with their targets are required for improved therapeutic antibodies[13]. Understanding the role of specific residues is an important factor in antibody rational design and engineering. This information can be derived via analyzing the three-dimensional structure of the antibody molecule or, when not available, via building and analyzing its three-dimensional model. Computational methods for *de novo* design of a fully human antibody against any specific antigen provide a route to resolve these issues[14]. Next to these experimental approaches are theoretical methods such as emerging bioinformatics tools to study protein complexes at structural levels based on docking[15]. Production of desirable antibodies is possible through antibody engineering by site-directed mutagenesis[15]. Also, prediction of favored sequences for engineering V domains is facilitated by bioinformatics tools.

Ofatumumab (anti-CD20 mAb) was approved in 2009 for the treatment of chronic lymphocytic leukemia. It is believed that Ofatumumab acts through immune-mediated mechanisms, particularly complement-dependent cytotoxicity, antibody-dependent cellular cytotoxicity by natural killer cells, and antibody-dependent phagocytosis by macrophages. It also enhances apoptosis[16].

Considering the importance of antibody engineering, we aimed to extend our knowledge on functionally important residues of ligand-binding site in Ofatumumab antibody variable region through proABC (prediction of antibody contacts) methodology[17]. High affinity variants of antibody were selected according to the results of docking programs[18] and molecular dynamics, which is a computer simulation method for studying the physical movements of atoms and molecules[19].

## MATERIALS AND METHODS

### Antigen and antibody sequences

CD20 amino acid sequence was obtained from UniProt at http://www.uniprot.org/ with NCBI accession number of P11836 at and from Protein Data Bank at https://www.rcsb.org with PDB ID.1S8b. Ofatumumab antibody sequence was extracted from IMGT/V-QUEST server (https://www.imgt.org/IMGT _vquest/vquest) and FPO (http://www. Freepatents online.com/) as well as from Protein Data Bank with PDB ID 3GIZ.

### Ofatumumab complementarity-determining region prediction

The Paratome web server[20] (http://ofranservices.biu.ac.il/site/services/paratome) was used for CDR prediction. This server can predict the antigen-binding regions (ABRs) of a given antibody, regarding its amino acid sequence or 3D structure. Paratome was built by structurally aligning a non-redundant set of all recognized antibody-antigen complexes in the PDB, from which structural consensus elements commonly involved in antigen binding to antibodies were identified.

### Ofatumumab interfaces prediction

PIER web server[21] (http://abagyan.ucsd.edu/PIER/) was used for Ofatumumab interface prediction. This server provides a tool to predict interfaces from a single protein structure on the basis of local statistical properties of the protein surface derived at the level of atomic groups. The proposed *PredUs software* (https://bhapp.c2b2.columbia.edu/PredUs/) is a flexible, interactive, template-based web server that uses *structural information* to predict what residues on protein surfaces are likely to participate in complexes with other proteins[22].

### Ofatumumab pocket and binding sites

GHECOM (grid-based HECOMi finder; http://strcomp.protein.osaka-u.ac.jp/ghecom/) is a software for finding multi-scale pockets on protein surfaces using mathematical morphology[23]. CASTp server[24] (http://sts.bioe.uic.edu/castp/) uses the weighted Delaunay triangulation and the alpha complex for shape measurements. It provides identification and measurements of surface accessible pockets as well as interior inaccessible cavities, for proteins and other molecules.

### Identification of functionally and structurally important residues in Ofatumumab antibody

Antibody sequence was used as an input file in ConSeq[25] (http://conseq.tau.ac.il/) to determine conserved functional and structural amino acids. The software parameters were set as follow: PSI-BLAST for five iterations against UniProt database with *E*. value of 0.01 and maximum likelihood, as a method of calculating amino acid conservation score. The proABC is a web server for predicting the residues in antibody-binding site, which are involved in antigen recognition (http://www.biocomputing.it/proABC).

### Antibody variants sketching and amino acid substitution

The amino acids located in the binding site of antibody were identified based on the results obtained from proABC, PIER, and ConSeq web applications. In this step, the suitable amino acid was selected for mutation design.

### Significant residue selection

Some amino acids were selected as significant residues in Ofatumumab structure by employing the results of different software. The selected residues in terms of PIER software have a score above 30, but in terms of Cons-PPISP software (http://pipe.scs.fsu.edu/ppisp.html), they have a score above 0.5. These residues located in one of the three CDR regions were predicted by Paratome server and confirmed by proABC software as an interactive amino acid.

### SIFT analyses and Ofatumumab 3D structure

SIFT server[26] were used to predict (http://sift.jcvi.org/) whether an amino acid substitution affects protein function. SIFT prediction is based on the degree of conservation of amino acid residues in sequence alignments derived from closely related sequences, collected through PSI-BLAST. The 3D structure of all offered variants was determined by PIGS server (http://circe.med.uniroma1.it/pigs/), which performs the automatic prediction of immunoglobulin variable domains based on the canonical structure model. The server is user-friendly and flexible. It allows the user to select templates for the frameworks and the loops using different strategies[27]. The final output is a full-fledged 3D model of the variable domains of the target immunoglobulin. Geometry optimization was performed on antibody modeling derived from PIGS web server using HyperChem 8.0 Professional software[28] to further improve the predicted structures.

### Protein-protein docking scrutiny

Docking was performed using Hex 8.0 software, ClusPro 2.0 server[29] cluspro (https://cluspro.bu.edu/publications.php), ZDOCK (http://zdock.umassmed. edu/)[30], and HADDOCK[31] at http://haddock.science.uu.nl/services/HADDOCK 2.2/. This scrutiny was used to determine the interaction and orientation between the two molecules to determine the correct binding between the antigen and the antibodies. These software was selected from those considered in the CAPRI project[32] (http://www.ebi.ac.uk/msd-srv/capri). The prediction of the interactions of structures was to use the main software setup, with the explanation that was used in ClusPro software using antibody prediction method.

### Two-dimensional representations of protein-ligand complexes

The LIGPLOT software (https://www.ebi.ac.uk/thornton-srv/software/LIGPLOT/) automatically generates schematic 2D representations of protein-ligand complexes from standard Protein Data Bank file input. The program is completely general for any ligand and can also be used to show other types of interaction in proteins and nucleic acids[33]. We calculated non-bonded contacts, hydrogen bonds, and hydrophobic interactions for all the complexes using the software LIGPLOT with default parameters.

### Molecular dynamics simulation of antibody variants–Cd20 complex

After identifying the mutated structure with the highest affinity to CD20 and correct binding orientation, the molecular dynamics was performed using GROMACS 4.6.5 and the control antibody. In this study, for molecular dynamics, pdb file format was used. These data were analyzed including an initial cubic salvation with a three-point simple water model, followed by ionization and neutralization of simulation cube with Na and Cl ions. Geometry optimization was performed with a constrain method. This procedure continued with two separate temperatures and pressure unconstrained global dynamics. Final unconstrained dynamics were performed with coupled temperature (300°K) and pressure (1 bar) for 20 ns[34].

## RESULTS

### Antigen and antibody sequence availability from FPO and databank

CD20 is a 297-amino-acid phosphoprotein with 4 transmembrane domains belonging to MS4A family, which their coding genes are located on the long arm of chromosome 11[35]. The Ofatumumab antibody contains 122 and 107 amino acids in the variable region of heavy and light chains, respectively.

### The prediction of Ofatumumab antigen-binding regions

ABRs in Ofatumumab[17] was predicted using Paratome. This server predicted six regions as ABRs in Ofatumumab sequences, three of which in light chains and three ones in heavy chains.

>paratome_1_VH (heavy chain)

ABR1: FTFNDYAMH (27-35)

ABR2: WVSTISWNSGSIGY (47-60)

ABR3: KDIQYGNYYYGMDV (98-111)

>paratome_1_VL (light chain)

ABR1: QSVSSYLA (27-34)

ABR2: LLIYDASNRAT (46-56)

ABR3: QQRSNWPI (89-96)

### Ofatumumab interface prediction

PIER server calculated a value for each residue. PIER value indicates how likely a certain residue is involved into a protein interface formation, with higher values indicating higher probability[21]. PIER values greater than 30 indicate very likely interface residues, and the values less than 0 specify very unlikely interface residues. PIER results are shown in Table 1.

**Table 1:** PIER results

IX	Molecule	Residue	PIER
32	L	Y32	34.16
96	L	I96	48.72
91	L	R91	49.94
100	H	I100	38.28
101	H	Q101	52.50
102	H	Y102	41.57
103	H	G103	49.04
104	H	N104	48.54
105	H	Y105	41.85
106	H	Y106	38.71
107	H	Y107	42.28

PIER server calculated a value for each residue. The Table indicates residues that have the value above 30.

### Ofatumumab pocket and binding site detection

**Fig. 1:**
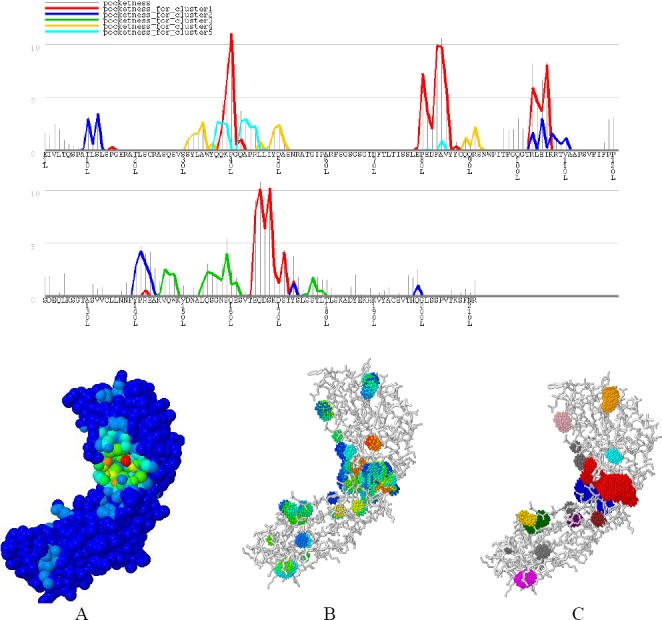
GHECOM results. Top: graph residue-based pocketness. The height of the bar shows the value of pocketness (%) for each residue. The color of pocketness bar indicates the cluster number of pocket: red, cluster 1; blue, cluster 2; green, cluster 3; yellow, cluster 4; cyan, cluster 5. Below: Jmol view of pocket structure based on (A) pocketness color, (B) depth color, and (C) cluster color. Biologically important functional residues annotated from three sources were mapped to PDB structures, and visualization was provided. The Figure shows the atoms of the charge relay system that resides in a functional pocket of Ofatumumab. CASTp server predicts functional areas in protein structure. This server measures analytically the area and volume of each pocket and cavity, both in a solvent accessible surface (Richards’ surface) and molecular surface (Connolly’s surface).

**Fig. 2:**
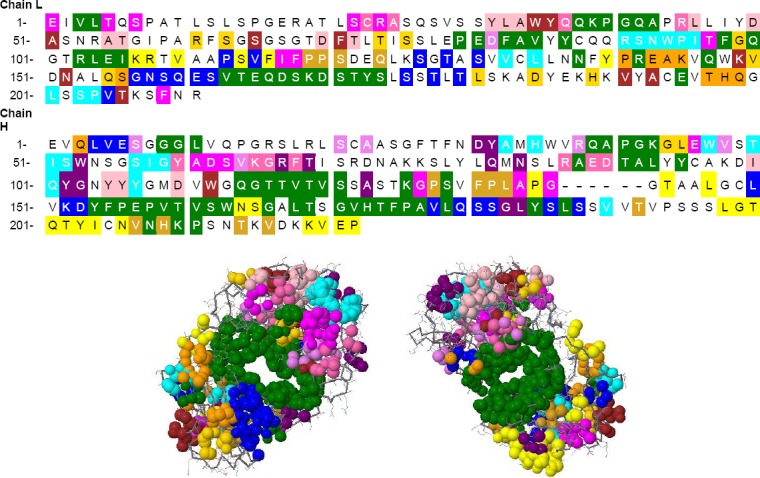
CASTp results. Residues are colored based on the area and volume size. The most important residue is illustrated in green, and other residues are shown in blue, cyan, yellow, magenta, pink, orange, purple, brown, gold, violet, hot pink, and gold, respectively.

GHECOM server found five pocket clusters on Ofatumumab surface using mathematical morphology. In this regard, GHECOM computes a pockets score (sum of 1/[Rpocket]/(1/[Rmin]*[vol of shell])) for each residue. A residue in a deeper and larger pocket has a larger value of pockets. The pockets of small-molecule binding sites and active sites were higher than the average value, specifically the values for the active sites were much higher. This result suggests that pockets contribute to the prediction of binding sites, and active sites from protein structures. GHECOM and CASTp results are shown in Fig. 1 and 2.

### Identification of functionally and structurally important residues in Ofatumumab antibody

**Fig. 3:**
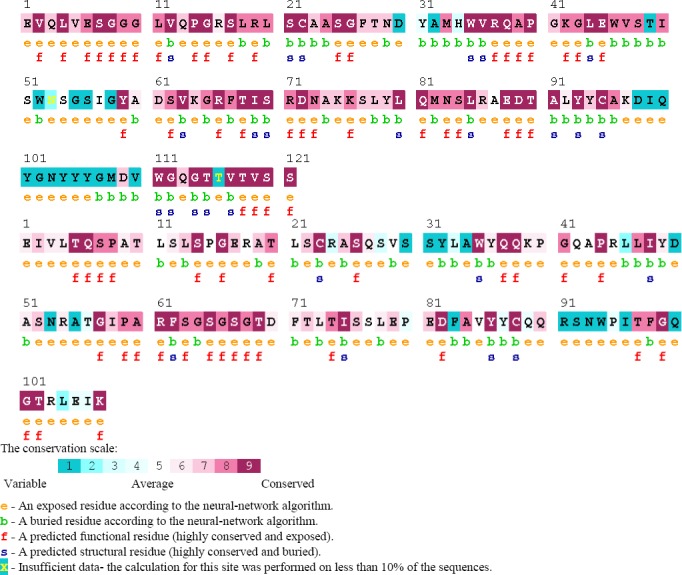
ConSeq results for identification of functionally and structurally important residues.

**Fig. 4:**
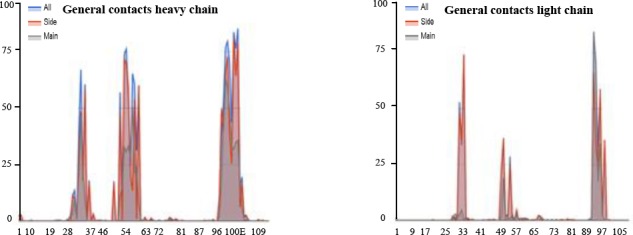
proABC results. proABC estimates, for each residue in its sequence, the interaction probability with the cognate antigen.

ConSeq annotated functional residues on sequence and structure of Ofatumumab antibody in twilight zones, respectively (Fig. 3). ProABC computations are based on a machine learning method trained on sequence and sequence-derived features. Starting from the antibody sequence alone, proABC estimates the interaction probability with the cognate antigen for each residue of the antibody (Fig. 4).

### Antibody variants sketching and amino acid substitution

The results from proABC, PIER, and ConSeq web applications indicated that the amino acids located in the binding site of antibody can interact with CD20. Mutations for variants 1-4 are as follows: (D98H/I), (I99H/W), (Q100H/L), (Q100H/L) for variant 1, ((Y32L/H), (R91L/G), (S92L/W), (N93L/G) variant 2, (Y32L/H ), (R91L/G), (S92L/W), (N93L/G), (D98H/I), (I99H/W), (Q100H/L), (Q100H/L), (G102H/V) for variant 3, and (R91L/G), (S92L/W), (N93L/G), (Q100H/L), (G102H/V) for variant 4.

### Protein-protein docking scrutiny

**Fig. 5:**
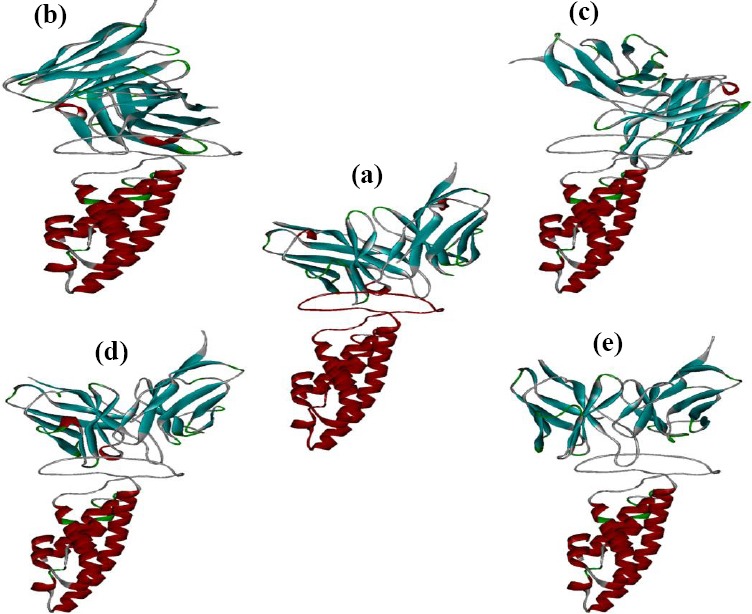
Docked positions indicated using HEX8.0 software. Interaction of (a) wild antibodies, (b) variant 1, (c) variant 2, (d) variant 3, and (e) variant 4.

In this study, HEX8.0 software, ClusPro ZDOCK, and HADDOCK were used for docking. These software perform docking of Ofatumumab antibody variants and CD20 antigen, completely flexible, using molecular dynamics simulations. In addition, based on biochemical and/or biophysical information, they search all possible binding modes in the translational and rotational space between the two proteins and evaluate each pose using an energy-based scoring function, root mean square deviation (RMSD), and interacting position. The desired variants were selected. The docking images of antibodies with antigen are presented in Figure 5. To calculate RMSD obtained from HEX8.0 software, Discovery Studio Visualizer v2.5.5.9350 was used.

Results of RMSD between the normal and mutated antibody variants with CD20 for variants 1, 2, 3, and 4 are 19.894, 20.667, 2.367, and 9.086, respectively. The results of docking between the normal and mutated antibody variants with CD20 antigen are shown in Table 2. Ranking of complex structure is based on HADDOCK scores. In this Table, the Van der Waals and electrostatic energy values as well as the buried surface between two complexes are shown. To calculate the binding energy between antibody molecules and CD20, PDBe Pisa software (http://www.ebi.ac.uk/pdbe/pisa/) was used; binding energy between antibody molecules and CD20 using PDBePISA software variant 1, 2, 3, and 4 and wild type antibody was reported as -13.6, -11.8, -15.4, -13.1, and -12.6, respectively.

**Table 2:** Results of docking between the normal and mutated antibody variants with CD20 antigen

Variants Feature	Control	Variant 1	Variant 2	Variant 3	Variant 4
HADDOCK score	-111.8 ± 9.4	-162.4 ± 2.2	-158.7 ± 8.8	-147.1 ± 3.9	-172.7 ± 11.9
Cluster size	7	53	110	11	11
RMSD from the overall lowest-energy structure	9.2 ± 0.2	0.5 ± 0.3	1.1 ± 0.7	10.7 ± 0.3	0.7 ± 0.5
Van der Waals energy	-67.1 ± 8.0	-67.8 ± 6.6	-69.2 ± 3.9	-63.1 ± 1.5	-83.4 ± 3.1
Electrostatic energy	-122.2 ± 17.9	-216.3 ± 24.1	-207.7 ± 23.7	-129.6 ± 37.5	-126.2 ± 24.4
Desolvation energy	-23.9 ± 4.1	-59.9 ± 6.5	-54.4 ± 6.7	-69.2 ± 9.7	-72.8 ± 7.8
Restraints violation energy	36.7 ± 4.65	84.8 ± 40.29	63.7 ± 11.17	111.9 ± 22.20	87.5 ± 63.92
Buried surface area	1828.6 ± 92.9	2102.0 ± 128.3	1948.7 ± 62.6	1981.4 ± 118.2	2351.7 ± 53.9
Z-Score	-2.2	-1.9	-2.0	-1.5	-1.2

### Two-dimensional representations of protein-ligand complexes

**Fig. 6:**
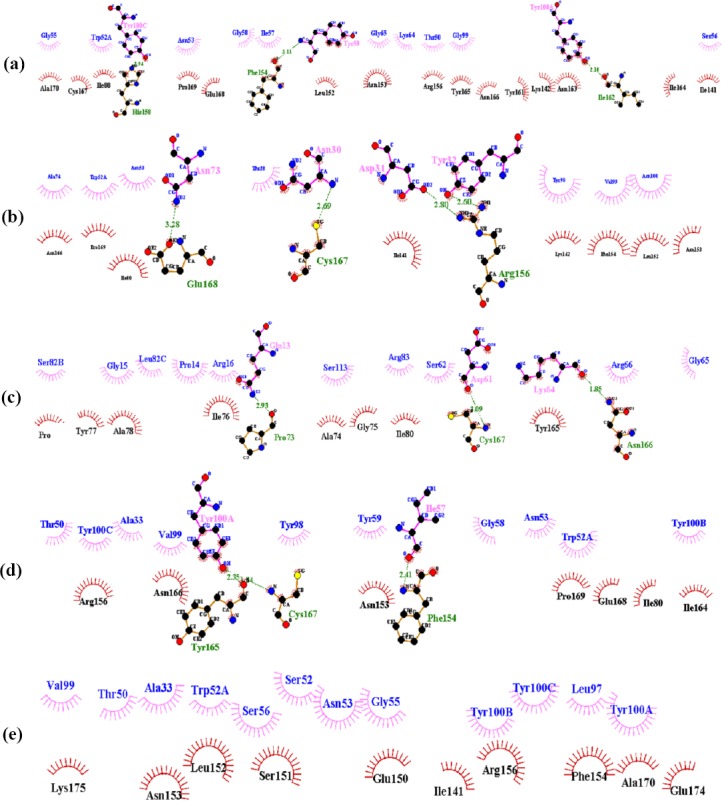
Docked positions indicated using LIGPLOT software. The plots report the non-bonded contact probability for each residue and separately, for its side chain and its main chain. Interaction of (a) wild antibodies, (b) variant 1, (c) variant 2, (d) variant 3, and (e) variant 4.

A 2D analysis of the amino acid of antibodies and antigens was performed in a precise and quantitative manner using the LIGPLOT server. The result of this software is presented in Figure 6.

### Molecular dynamics simulation of antibody variants-Cd20 complex

**Fig. 7:**
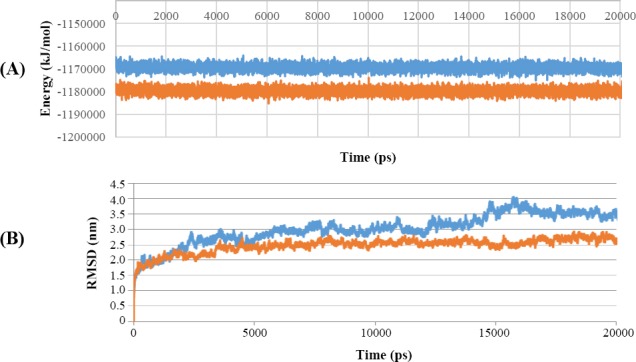
Molecular dynamics simulation result. (A) Energy plots for the molecular dynamics simulation. (B) RMSD plot. The lower line (orange) is related to the mutated structure, while the upper line (blue) is related to the normal structure.

Molecular dynamics simulation was performed using GROMACS software for 20 multiple-nanosecond time scale. Molecular dynamics is required to ensure the stability of antibodies and antigen complex. Total energy and the RMSD plot were conserved by maintaining the temperature and pressure levels during trajectory monitoring. The result of this software is presented in Figure 7.

## DISCUSSION

The significance of mAbs is increasingly rising in various areas, including industry, medicine, biosensor design, and basic research. In the last two decades, the mAb therapy has emerged as one of the most promising approaches of biological therapeutics[36,37]. The unique molecular structure of antibodies facilitates their bivalent binding to a broad variety of antigenic epitopes such as proteins, carbohydrates as well as nucleic acids. As a result, antibodies can be used as research, diagnostic, and therapeutic reagents[38,39].

Several key characteristics of mAbs should be optimized in order to use them as therapeutic agents, including binding affinity and specificity, folding stability, solubility, pharmacokinetics, effector functions, and the capability of binding to additional antibody domains (bispecific antibodies) or cytotoxic drugs (antibody-drug conjugates). Optimization strategies for mAbs provide the possibility of modification and improvement of an antibody molecule in nearly all clinically relevant aspects, but the experimental procedures are expensive and time-consuming. Besides, the use of systematic design methods is needed to overcome these and other challenges in order to complement powerful immunization and *in vitro* screening methods. One of the most important features of antibodies is their capability to recognize targets with high affinity and specificity.

Since the interactions of antigen-antibody take place at the atomic levels, characterization of antibody structure and the properties of its binding site are beneficial to understand their mechanisms of actions in order to design improved antibodies. In spite of their benefits, mAbs originated from both human and xenogeneic sources have several defects, including short *in vivo* life, low stability, and high probability to raise an immunogenic reaction in patients. Several strategies, based on genetic recombination, have been developed and optimized to overcome these obstacles. However, understanding the structure and binding mode of the specific antibody may accelerate optimization of antibody[40].

The binding activity of the antibody is mainly mediated through complementarity-determining region (CDRs). Different kinds of novel techniques have been developed regarding the design of CDRs[41]. Because of the complexity of *de novo* design methods, researchers have tried to use design methods in order to improve the binding affinity of available antibodies. Antibody engineering methods are important and more considered because the low affinity antibodies, which do not meet the therapeutic applications, are commonly developed after immunization. Also, the need for unrealistically large libraries restricts the ability of directed evolution strategies to identify multiple synergistic mutations. In this regard, interesting studies performed to improve antibody affinity demonstrated the potential of optimizing electrostatic inter-actions[42,43]. Different methods for designing antibodies are increasingly being used to decrease the reliance on screening and immunization in order to improve important antibody characteristics. Combinatorial and computational methods, alone or in combination, may be employed to optimize the binding properties of natural protein-protein interactions for various biomedical and synthetic biology applications[44].

Considering the benefits of Ofatatmumab antibody and the potential impact of affinity improvement on its efficacy, biological activity, and treatment costs, we, in the present study, aimed to evaluate *in sillico* modeling approach to improve binding affinity of this antibody. We also used the Paratome web server to predict the ABRs of a given antibody, according to its amino acid sequence or 3D structure. This server predicted six regions as ABRs in Ofatumumab sequences, three in light chains, and three ones in heavy chains. FTFNDYAMH[27-35] as ABR1, WVSTISWNSGSI GY[47-60] as ABR2, and KDIQYGNYYYGMDV (98-111) as ABR3 as well as three regions in heavy chains include QSVSSYLA[27-34] as ABR1 and LLIYDASNRAT[46-56] as ABR2, and QQRSNW PI[89-96] as ABR3. Following prediction of ABRs with Paratome web server, we used this server to predict interfaces from a single protein structure. PIER server calculated a value for each residue. PIER value indicates how likely a particular residue is involved in a protein interface formation, with higher values, indicating higher probability. Y32L, R91L, S92L, N93L, D98H, I99H, Q100H, and G102H residues have PIER value above 30.

Identification of functionally and structurally important residues in Ofatumumab antibody was predicted by proABC server. This server estimates the interaction probability of each residue with the cognate antigen. Also, ConSeq server predicted several residues, which were identified as functional residues and considered highly conserved and exposed. proABC server predicted Y32L, R91L, S92L, N93L, D98H, I99H, Q100H, and G102H as functionally important residues in Ofatumumab antibody. Paratome, PIER, and ConSeq as well as proABC server results demonstrated some functional conserved residues. It seems that these amino acids are involved in CD20 antigen to antibody interactions (Table 1).

In this study, antibody modeling and geometry optimization were performed using the PIGS web server and HyperChem software, respectively. This approach led to a reduction in the energy of all structures and, as a result, the final structures of the coordinates were similar to the structure of Ofatumumab antibody. Based on these findings, we replaced amino acids and then conducted docking, which plays an important role in rational and targeted drug design because this is the only computational approach that directly models physical interactions between proteins. Docking is capable of predicting complexes of modeled proteins in molecular modeling. Docking refers to the way in which the orientation and mode of binding of one molecule to another is predicted during the formation of a stable complex[45]. We used HEX8.0 software, ClusPro, ZDOCK, and HADDOCK for docking Ofatumumab variants and CD20 antigen.

Data from a previous study was used to determine which software performs better predictions. Based on the x-ray crystallography[45], the Ofatumumab antibody that interacts with both loops on the CD20 antigen contains amino acids 79-84 and 142-188. Among the software used, HADDOCK and HEX8.0 predicted the best and most similar interactions with existing data between antibodies and antigens; however, the HEX8.0 showed better results. Another criterion for choosing better interactions was the predicted interaction with the membrane. Thus, the predictions in which antibodies interact with the membrane portion of the antigen were ignored.

The results of docking with HEX8.0 software for both normal and mutant antibodies suggest that designed mutations have improved the binding and energy properties to the antigens in mutated antibodies compared to original antibody (Fig. 5). There are two criteria for comparison of docking results. The first criterion is the RMSD, between the mutated types and the control. RMSD[46] is a measure that shows the degree of structural similarity between mutated types and the control structures. The similarity of the two structures is higher. The second criterion is the amount of energy (ΔG). The smaller energy complexes have the greater stability and the greater binding affinity. Based on the results obtained from RMSD, variant 3 antibodies has smaller RMSD amount, then it has most similarity in orientation of the binding between wild antibodies and CD20. Amount of bonding energy and its stability has also been increased compared to the wild type.

LIGPLOT software was conducted to ensure that the variant 3 antibody is selected correctly. Results from LIGPLOT are shown in Figure 6. Variant 3 antibodies have the highest number of hydrophobic interactions and shorter hydrogen bonds, which indicates a greater affinity to Cd20 antigen. On the other hand, the type of amino acids involved in interactions between variant 3 antibodies and CD20 has more commonality with the wild type, which again shows the similarity of their binding orientation. All these results suggest that variant 3 mutations have improved the characteristics of antibody binding compared to normal antibodies.

The molecular dynamics simulation results were analyzed based on total energy and RMSD. Energy results indicated that the binding of antibody and antigen complexes over the relevant time period is stable. Energy and RMSD have not been posed in the given time interval and have not been molecular disintegration (Fig. 7).

Total energy diagram shows that during molecular dynamics study, the mutated structure has a lower energy content and therefore has a higher stability than the normal antibody. Since part of this energy is related to the binding energy, it can be concluded that binding energy in the mutated complex is lower, and there is a greater affinity between mutant antibody and antigens.

Antibodies are proteins that are the recognition elements of the immune system and increasingly used as drugs in cancer therapy. A growing number of antibodies are being applied for the treatment of leukemia with high success. The application of technologies, such as phage display, for the preparation of human antibodies has paved the way for this success. However, the initial affinities of these antibodies are typically too low for therapeutic application. High affinity and selectivity are critical issues for antibody therapeutic capacity. Rational engineering methods can be applied with reasonable success to optimize physicochemical characteristics of antibodies. The aim of the computational design is to generate antibodies at desired affinity, specificity, and half-life.

In this study, our findings showed that variant 3 mutations have improved the characteristics of antibody binding compared to normal Ofatumumab antibodies. Therefore, data reported in this paper represents the first step toward development of a new anti-CD20 antibody against B-Cell malignancy.
